# The effects of cucurbitacin E on GADD45β‐trigger G2/M arrest and JNK‐independent pathway in brain cancer cells

**DOI:** 10.1111/jcmm.14250

**Published:** 2019-03-25

**Authors:** An‐Chin Cheng, Yi‐Chiang Hsu, Chiang‐Chin Tsai

**Affiliations:** ^1^ Department of Nutrition and Health Sciences College of Health Sciences Chang Jung Christian University Tainan Taiwan; ^2^ Department of Medical Sciences Industry College of Health Sciences, Chang Jung Christian University Tainan Taiwan; ^3^ Department of General Surgery Tainan Sin Lau Hospital, The Presbyterian Church in Taiwan Tainan Taiwan; ^4^ Department of Health Care Administration College of Health Sciences Chang Jung Christian University Tainan Taiwan

**Keywords:** cucurbitacin E, GADD45β, glioblastoma‐astrocytoma, glioma, mitosis delay

## Abstract

Cucurbitacin E (CuE), an active compound of the cucurbitacin family, possesses a variety of pharmacological functions and chemotherapy potential. Cucurbitacin E exhibits inhibitory effects in several types of cancer; however, its anticancer effects on brain cancer remain obscure and require further interpretation. In this study, efforts were initiated to inspect whether CuE can contribute to anti‐proliferation in human brain malignant glioma GBM 8401 cells and glioblastoma‐astrocytoma U‐87‐MG cells. An MTT assay measured CuE's inhibitory effect on the growth of glioblastomas (GBMs). A flow cytometry approach was used for the assessment of DNA content and cell cycle analysis. DNA damage 45β (GADD45β) gene expression and CDC2/cyclin‐B1 disassociation were investigated by quantitative real‐time PCR and Western blot analysis. Based on our results, CuE showed growth‐inhibiting effects on GBM 8401 and U‐87‐MG cells. Moreover, GADD45β caused the accumulation of CuE‐treated G2/M‐phase cells. The disassociation of the CDC2/cyclin‐B1 complex demonstrated the known effects of CuE against GBM 8401 and U‐87‐MG cancer cells. Additionally, CuE may also exert antitumour activities in established brain cancer cells. In conclusion, CuE inhibited cell proliferation and induced mitosis delay in cancer cells, suggesting its potential applicability as an antitumour agent.

## 1 INTRODUCTION

A previous study reported that brain cancer is one of the most invasive and malignant cancers in developed countries.^1^ However, the chemotherapy for brain cancer remains obscure. Two of the best strategies for tumour suppression are the induction of apoptosis (type I cell death) and cell cycle arrest in cancer cells.^2^ Recent studies have demonstrated that phytochemicals synthesized from plants can suppress tumour growth by inducing apoptosis and cell cycle arrest, and down‐regulating gap junctional intercellular communication.^3^, ^4^, ^5^

Cucurbitacins, a class of tetracyclic triterpenoids with medicinal properties in Cucurbitaceae, are extracted from the climbing stems of *Cucumis melo* L.^6^ Cucurbitacins have been widely used in inhibition of cancer cell progression as medicinal herbs throughout Asia.^7^ In recent years, there is a growing interest in this herb because of its presumed beneficial pharmacological properties as anti‐inflammatory^8^ and antitumour agents.^9^

Cucurbitacin E (CuE) is an active compound of the cucurbitacin family.^10^ Recent reports have demonstrated that CuE possesses various pharmacological functions, such as antiviral, anti‐inflammatory and anticancer effects.^11^, ^12^, ^13^ Cucurbitacin E exhibits inhibitory effects in several types of cancer 10, 14; however, its anticancer effect in brain cancer remains unclear. Therefore, the mechanism underlying the antitumour effect of CuE on brain cancer has yet to be identified.

Glioblastomas (GBMs) are highly invasive and recurrence brain tumours,^15^ and have been shown to harbour therapy‐resistant cancer stem cells (CSCs), and this is the main cause of death.^16^, ^17^ Recent studies indicated that GBMs contain a subpopulation of glioma‐initiating tumour cells which exhibits stem cell characteristics and may be responsible for in vivo tumour growth.^18^, ^19^ Therefore, we chose the GBM 8401 and glioblastoma‐astrocytoma U‐87‐MG cells as human brain cancer model to analyse the antitumour activity of CuE.

In the current study, efforts have been initiated to inspect whether CuE can contribute to the anti‐proliferation of GBM 8401 and U‐87‐MG cells. Apoptosis^20^ and cell cycle regulation^21^ have been posited as possible targets for cancer therapy, and CuE was found to induce the regulation of cell cycle progression.^22^ Therefore, we focused specifically on the effects of CuE on the induced delay of mitosis and gene expression in GBM 8401 and U‐87‐MG cells. We expect that our study may provide a scientific foundation and technological support for brain GBM therapy.

## 2 MATERIALS AND METHODS

### 2.1 Materials

All reagents and chemicals were of analytical grade. Cell culture materials including DMEM, Roswell Park Memorial Institute medium (RPMI), foetal bovine serum (FBS), phosphate‐buffered saline (PBS), sodium pyruvate, antibiotics and trypsin were purchased from Gibco, BRL (Grand Island, NY). Cucurbitacin E, dimethyl sulphoxide (DMSO) and 3‐(4,5‐dimethylthiazol‐2‐yl)‐2,5‐diphenyltetrazolium bromide (MTT) were purchased from Sigma (St. Louis, MO). Annexin V‐FITC was from BD Pharmingen (San Diego, CA, USA), D‐tetripeptide 3 (DTP3) was from Sigma (St. Louis, MO, USA), molecular weight markers were from Bio‐Rad and polyvinylidene fluoride (PVDF) membranes were purchased from Millipore Merck (Darmstadt, Germany).

### 2.2 Cells

Human glioblastoma‐astrocytoma U‐87‐MG (NCI‐PBCF‐HTB14; ATCC HTB‐14) and human brain malignant glioma GBM 8401 cells were obtained from Bioresource Collection and Research Center (BCRC, Hsinchu, Taiwan). GBM 8401 cells were maintained in 90% (v/v) RPMI 1640 and 10% (v/v) FBS with 2 mmol/L l‐glutamine and 1.5 g/L sodium bicarbonate, and U‐87‐MG cells were maintained in DMEM with 10% (v/v) FBS with 2 mmol/L l‐glutamine, 1.5 g/L sodium bicarbonate and 0.1 mmol/L non‐essential amino acid (NEAA). These cells were incubated in a humidified atmosphere of 5% CO_2_ and 95% air at 37°C.

### 2.3 Cell proliferation assay

Cells were seeded into a 96‐well culture plate at 5000 cells/well followed by the addition of 0, 2.5, 5 or 10 μmol/L CuE for 1‐3 days. 3‐(4,5‐Dimethylthiazol‐2‐yl)‐2,5‐diphenyltetrazolium bromide (1 mg/mL) was added to each well for the last 4 hours, and the reaction was blocked by adding DMSO and measured at 490 nm using a multiwell plate reader BioTek (Taipei, Taiwan).

### 2.4 Apoptosis measurement

The cells were plated in six‐well culture plates (Orange Scientific, EU). The cells were harvested and centrifuged after the incubation with CuE for 4 hours, and the cell pellet was then resuspended in 1 × annexin‐binding buffer containing 5 μL of annexin V‐FITC and 1 μL of 100 μg/mL propidium iodide (PI) and incubated for 15 minutes at room temperature. The stained cells were detected by a FACSCalibur flow cytometer (BD Pharmingen) and analysed using WinMDI 2.9 free software (BD Pharmingen).

### 2.5 Cell cycle analysis

A fluorescent nucleic acid dye PI was used to identify the proportion of cells in each interphase stage of the cell cycle. The cells were treated with CuE for 24 hours, and then harvested and fixed in 1 mL of cold 70% ethanol for at least 8 hours at −20°C. DNA was stained using a PI/RNaseA solution, and the DNA content was detected using a FACSCalibur flow cytometer. Data were analysed using WinMDI 2.9.

### 2.6 Mitotic index analysis

After appropriate treatment, the cells were harvested and fixed overnight in 70% ethanol. The cells were washed and suspended in 100 µL of IFA‐Tx buffer containing 4% foetal calf serum, 150 nmol/L NaCl, 10 nmol/L HEPES (siRNA) (4‐(2‐hydroxyethyl)‐1‐piperazineethanesulfonic acid), 0.1% sodium azide and 0.1% Triton X‐100 with the mitotic protein monoclonal 2 (MPM‐2) (anti‐phospho‐Ser/Thr‐Pro) antibody for 1 hour at room temperature followed by a rabbit antimouse Fluorescein isothiocyanate (FITC)‐conjugated secondary antibody (1:50 dilution; AbD Serotec (Kidlington, Oxford, UK) for 1 hour at room temperature in the dark. Finally, the cells were washed and resuspended in 500 µL of PBS with 20 µg/mL PI (Sigma) for 30 minutes in the dark. The expression of MPM‐2 was analysed on a FACSCalibur flow cytometer. Data were analysed using WinMDI 2.9.

### 2.7 Western blot analysis

Cell lysates (50‐75 µg) were loaded onto 10%‐12% SDS‐PAGE membranes and transferred to PVDF membranes (Millipore).The membranes were blocked overnight in Odyssey blocking buffer and incubated with anti‐DNA damage 45β (GADD45β) Abcam (Cambridge, UK), anti‐cell division cycle 2 (CDC2) (p34; sc‐747), anti‐ERK (SC‐94), anti‐phospho ERK (SC‐7383), anti‐JNK1/3 (SC‐474), anti‐cyclin B1 Santa Cruz Biotechnology (Dallas, TX, USA) or anti‐β‐actin (Sigma‐Aldrich) antibodies for 90‐120 minutes. The blots were washed and incubated with a corresponding secondary antibody Li‐COR (Taipei, Taiwan) at a 1:20 000 dilution for 30‐45 minutes. The antigens were then visualized using a near‐infrared imaging system Li‐COR, and the data were analysed using the software Odyssey 2.1 or a chemiluminescence detection kit (ECL; Amersham Corp., Arlington Heights, IL). Data were analysed using Odyssey 2.1 software.

### 2.8 Coimmunoprecipitation

Briefly, 500 μg of cellular proteins was labelled by anti‐CDC2 (p34; sc‐747), and the protein‐antibody immunoprecipitates were collected using protein A/G plus‐agarose beads (SC‐2003; Santa Cruz BioTechnology). DNA damage 45β protein levels was detected by Western blot analysis and visualized using a chemiluminescence detection kit (ECL; Amersham Corp.), and data were analysed using Odyssey 2.1 software.

### 2.9 Quantitative real‐time PCR

Quantitative real‐time PCR (qRT‐PCR) was performed with approximately 200 ng of SYBR Green PCR MasterMix in an ABI 7300 system (Applied Biosystems, Foster City, CA) under the condition of 40 cycles: 95°C for 120 seconds, 60°C for 30 seconds and 72°C for 30 seconds. To normalize readings, we used Ct values obtained at 18 seconds as internal controls for each run, obtaining a delta Ct value for each gene. All protocols were carried out in accordance with the manufacturer's instructions.

### 2.10 Small‐interfering RNA

The specific small‐interfering RNA (siRNA) of GADD45β (Level com, Taiwan) and Lipofectamine RNAiMAX gene transfection system were purchased from Invitrogen (Thermo Fisher Scientific, Waltham, MA, USA). The transfection protocol was in accordance with manufacturer's instructions.

### 2.11 Statistical analysis

All data are reported as the mean (±SEM) of at least three separate experiments. The *t* test or one‐way ANOVA with a Scheffe's post hoc test was used for statistical analysis, with significant differences determined at *P* < 0.05.

## 3 RESULTS

### 3.1 CuE inhibits the cell viabilities of GBM8401and U‐87‐MG cells

**Figure 1:**
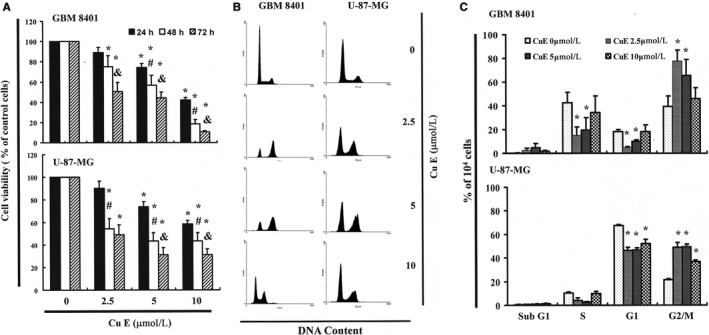
Cucurbitacin E (CuE) mediates the survival of GBM8401 and U‐87‐MG cells by inhibiting proliferation: (A) cells were treated with increasing doses of CuE (0, 2.5, 5 and 10 μmol/L) for 24‐72 h in vitro. The survival of CuE‐treated cancer cells was measured using the 3‐(4,5‐dimethylthiazol‐2‐yl)‐2,5‐diphenyltetrazolium bromide assay. Results are expressed as a percentage of the control, considered to be 100%. All data are reported as the mean (±SEM) of six separate experiments. Statistical analysis was performed using the *t* test, with differences between the treatment and control groups (0 μmol/L CuE) considered significant at *P* < 0.05, delineated by *. # and & *P* < 0.05 vs the 24 and 48 h. Effect of CuE on cell cycle progression and distribution in brain cancer cell lines: (B) cell cycle analysis of the cancer cells after being cultured with CuE for 24 h. C, Cucurbitacin E induced an increase in G2/M‐phase cell percentage (%). Cells underwent staining with propidium iodide to analyse DNA content, which was then quantified through flow cytometry. * in each group of bars indicates that the number of G2/M cells in the CuE treatment group was significantly higher than that of the control group (*P* < 0.05)

To investigate the antitumour effects of CuE on cell survival and proliferation, an in vitro study was applied to treat GBM8401 and U‐87‐MG cells with 0, 2.5, 5 and 10 μmol/L of CuE as indicated for 24‐72 hours. As shown in Figure 1A, the viabilities of GBM8401 and U‐87‐MG cells were significantly inhibited by CuE in a dose‐dependent pattern. Microscopic examination was used to observe morphological changes in the cells following 6‐24 hours of exposure to 5 μmol/L of CuE. Dead cell suspension in the medium indicated that CuE could induce cancer cell death (data not shown).

### 3.2 Non‐significant changes in apoptosis/necrosis in CuE‐treated brain cancer cells

To clarify the apoptosis/necrosis effects of CuE on the brain cancer cells, the cells were treated with CuE for 4 hours followed by detecting the generation of sub‐G1 cells by Annexin V‐FITC and PI staining, and the apoptotic ratios were quantified with flow cytometry. Flow cytometry dot‐plots of Annexin V‐FITC/PI staining illustrated non‐significant changes in apoptosis or necrosis ratios in CuE‐treated cells compared with that of untreated (control) cells (Figure S1A,B). Moreover, no significant increase in the ratio of caspase‐3 activity (data not shown) was observed in CuE‐treated cancer cells.

### 3.3 CuE enhances the numbers cell populations in the G2/M phase

To further investigate the effect of CuE on GBM8401 and U‐87‐MG cell growth, the cell cycle distribution among CuE‐treated cells was analysed and quantified using flow cytometry. As shown in Figure 1B, treatment with CuE resulted in accumulation of cells in the G_2_/M phase, implying that the brain cancer cell lines underwent mitosis delay. Our observed delay in mitosis implied that exposure to CuE enhanced the cell number in the G_2_/M phase while synchronously decrease the cell populations in other phases (Figure 1C).

### 3.4 Effects of CuE on the mitotic index

**Figure 2:**
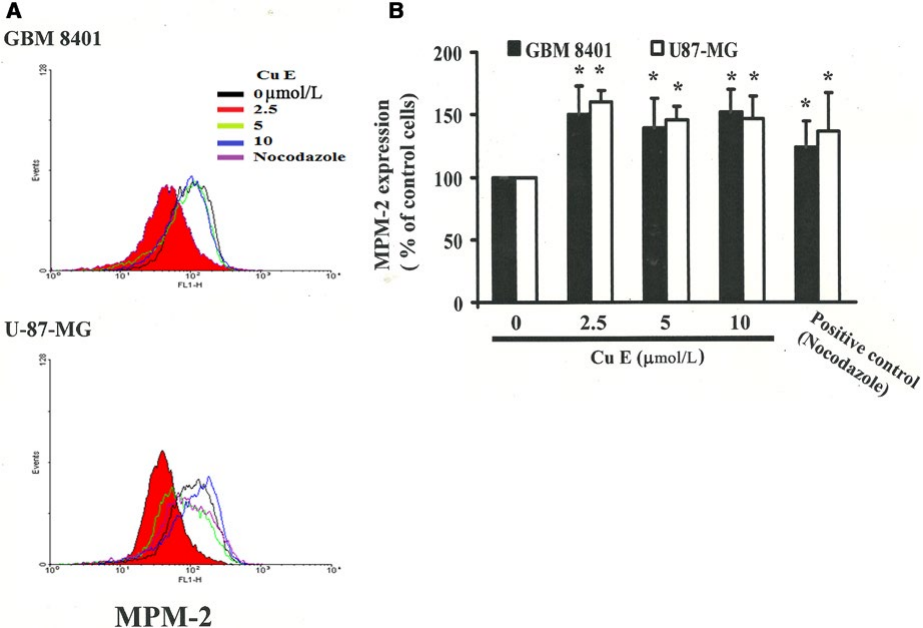
Diminished MPM‐2 activity in cucurbitacin E (CuE)‐treated GBM8401 and U‐87‐MG cells. A, Quantification of MPM‐2 expression (gated cells) was performed using flow cytometry following treatment with CuE for 24 h. B, Cucurbitacin E enhanced the level of MPM‐2 in those cells. As a positive control, separate groups of cells were treated for 24 h with Nocodazole (15 µg/mL), an antifungal agent that induces metaphase arrest. *in each group of bars indicates that the difference resulting from treatment with 0 μmol/L CuE was statistically significant at *P* < 0.05

MPM‐2 is a commonly used marker of mitotic disturbance that can bind to phosphorylated amino acid epitopes during mitosis, especially with maximum staining at the G2/M phase. To discriminate G2 arrest from mitotic arrest, we employed MPM‐2 (anti‐phospho‐Ser/Thr‐Pro)‐FITC (Figure 2A) as a mitosis‐specific antibody. The cells were treated with an inducer of metaphase arrest, nocodazole (15 µg/mL), for 24 hours as a positive control, which led to the synchronization of the entire cell populations in the G_2_/M phase and increased the MPM‐2 labelling (Figure 2B). MPM‐2 levels were increased in CuE‐treated brain cancer cell lines compared with those in the control group (Figure 2B). However, the effects of staining with MPM‐2 were similar to those of nocodazole in the CuE‐treated group. We suggest that cells were possibly stained with MPM‐2 in diverse stages of mitosis, and part of this result could not be identified by this early mitosis‐specific marker. In particular, part of the G_2_/M cells may remain unstained. Therefore, the increasing of MPM‐2 staining may imply underestimation of mitotic disturbance.

### 3.5 G_2_/M‐phase cell cycle arrest by CuE in GBM8401 and U‐87‐MG cells through CDC2 and cyclin B1disassociation with GADD45β up‐regulation

**Figure 3:**
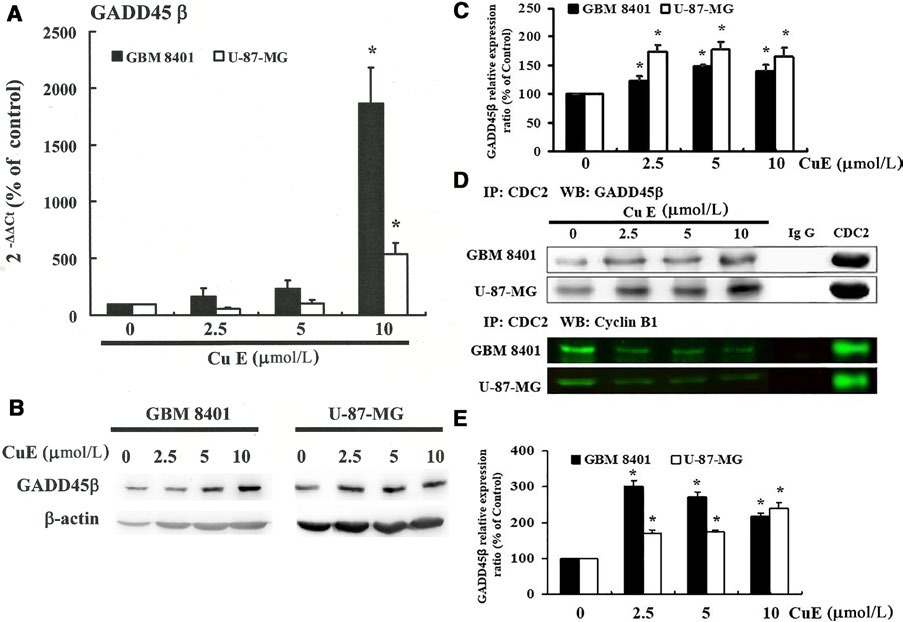
Cucurbitacin E (CuE) enhances DNA damage 45β (GADD45β) gene expression and CDC2/cyclin B1 disassociation in GBM8401 and U‐87‐MG cells. Cells were treated with CuE for 24 h; the gene and protein expression was subsequently detected using (A) q‐RT‐PCR and (B) Western blot analysis: (C) representative blots from three independent experiments and quantification of band intensities. D, Co‐immunoprecipitation of CDC2 and GADD45β and of CDC2 and cyclin B1 in brain cancer cell lines which treated with CuE. Non‐specific rabbit Immunoglobulin G (IgGs) was used as negative control; and CDC2 was used as an internal control. E, Quantification of band intensities. All data are reported as the mean (±SEM) of three separate experiments. Statistical analysis was performed using the *t* test, with differences considered significant at a level of *P < 0.05 vs the 0 μmol/L CuE control group

As shown in Figure 3, cellular proteins were extracted from the brain cancer cell lines treated with CuE, which was followed by qRT‐PCR, co‐immunoprecipitation (Co‐IP) and Western blot analysis. CDC2, cyclin B1 and GADD45β gene expression levels were quantified based on measurements of relative intensities. There was no significant change in mRNA levels following treatment with CuE (Figure S2A); in contrast, the protein expressions of CDC2 and cyclin B1 were significantly down‐regulated in CuE‐treated GBM8401 and U‐87‐MG cells (Figure S2B,C). Furthermore, Co‐IP test was performed to quantify the activities of GADD45β/CDC2 and cyclin B1/CDC 2, the critical roles for G_2_‐M transition during the cell cycle (Figure 3D). The results indicated that the increasing number of the brain cancer cell lines in the G_2_/M phase may via CuE‐induced association of CDC2 and the GADD45β complex (Figure 3E), and the disassociation of the CDC2/cyclin B1 complex in GBM8401 and U‐87‐MG cells by CuE can be attributed to GADD45β up‐regulation (Figure 3A‐C).

### 3.6 Reduction in the activity of GADD45β in brain cancer cells by DTP3 was reversed by CuE‐induced G_2_/M arrest

**Figure 4:**
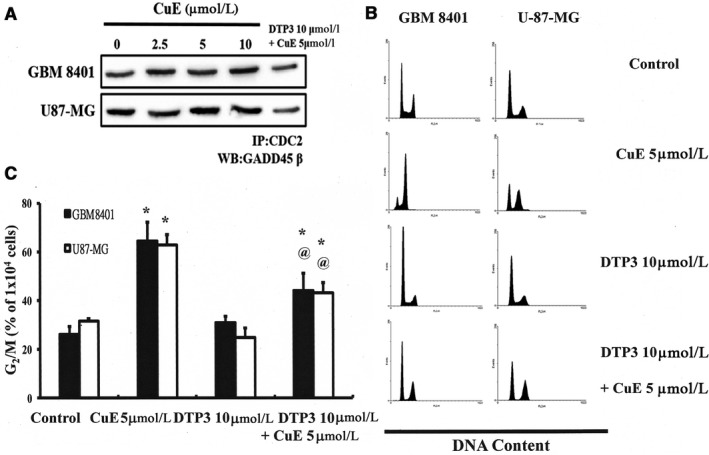
D‐Tetripeptide 3 (DTP3) represses DNA damage 45β (GADD45β) activity in GBM8401 and U‐87‐MG cells. Cells were treated with Cucurbitacin E (CuE), (A) GADD45β activity was subsequently detected using Co‐IP of CDC2 and GADD45β, (B) cell cycle analysis of the cancer cells after being cultured with CuE with or without DTP3, and (C) DTP3 repressed the CuE‐induced increase in G2/M‐phase cell percentage (%). Cells underwent staining with propidium iodide to analyse DNA content, which was then quantified through flow cytometry. All data are reported as the mean (±SEM) of three separate experiments. * in each group of bars indicates that the number of G2/M cells in the CuE treatment group was significantly higher than that of the control group (*P* < 0.05), delineated by ^@^ *P* < 0.05 vs the CuE 5 μmol/L group

Figure 4A illustrates the level of the GADD45β/CDC2 complex by Co‐IP and the immunoblotting results of cellular proteins in CuE‐treated brain cancer cell lines with or without DTP3 (GADD45β/MKK7 inhibitor). GADD45β was quantified based on measurement of relative band intensities. Figure 4A revealed that the expression of the GADD45β/CDC2 complex was repressed following incubation with CuE and DTP3 in those cancer cell lines.

To verify the connection between GADD45β up‐regulation and G2/M arrest in brain cancer cells, DTP3 was used to modulate the activity of GADD45β. Furthermore, we explored the effect of cellular GADD45β/CDC2 expression on cell growth‐inhibiting activity in GBM8401 and U‐87‐MG cells. The results revealed that the inhibition of GADD45β activity significantly suppressed CuE‐induced G_2_/M arrest in GBM8401 and U‐87‐MG cells (Figure 4B,C), implying that GADD45β could regulate the survival of GBM8401 and U‐87‐MG cells via CuE.

### 3.7 Down‐regulation of GADD45β in GBM8401 and U‐87‐MG cells by silencing GADD45β was reversed by CuE‐induced G_2_/M arrest

**Figure 5:**
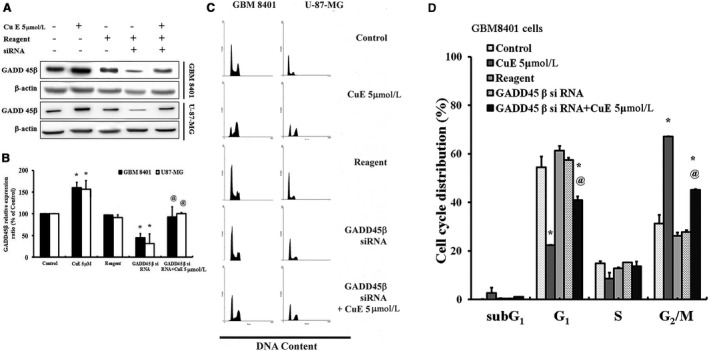
Inhibition of DNA damage 45β (GADD45β) could alleviate cell cycle arrest upon cucurbitacin E (CuE) treatment in GBM8401 and U‐87‐MG cells. A, Expression of GADD45β in brain cancer cells treated CuE and/or GADD45β siRNA. B, Representative blots and quantification of band intensities from three independent experiments. C, Cucurbitacin E delayed the progression of mitosis, whereas the addition of GADD45β siRNA could resume cell cycle. D, Cell cycle distribution (%) in GBM8401 cells. Cells underwent staining with propidium iodide to analyse DNA content, followed by quantification through flow cytometry. The control group was used as 100% (target gene/beta actin × 100%) to calculate the ratio of each group. All data are reported as the mean (±SEM) of three separate experiments. *P < 0.05 compared with control; ^@^ *P* < 0.05 compared with CuE 5 μmol/L

To verify the connection between GADD45β expression and G2/M arrest in GBM8401 and U‐87‐MG cells, Lipofectamine RNAiMAX gene delivery techniques were used to modulate cellular GADD45β levels, and the influences of GADD45β levels on cell growth‐inhibiting activity in GBM8401 and U‐87‐MG cells were investigated. Protein analysis revealed that GADD45β was significantly reduced after GADD45β was silenced (Figure 5A), whereas quantitative PCR analysis revealed that the higher expression levels of GADD45β mRNA in CuE‐treated brain cancer cells were significantly decreased after GADD45β was silenced (Figure 3A). Moreover, silencing GADD45β significantly suppressed CuE‐induced G2/M arrest in GBM8401 and U‐87‐MG cells (Figure 5C,D). The results illustrated that CuE could regulate the cell cycle progression of GBM8401 and U‐87‐MG cells via GADD45β.

The results from PCR array detection where a focused panel of genes was analysed by a RT^2^ Profiler™ PCR Array (PAHS‐020Z, QIAGEN; data not shown) were further confirmed by quantitative PCR analysis (Figure 3A; Figure S2), which indicated substantial GADD45β up‐regulation as well as significant down‐regulation of cyclin B, CDC2 and the GADD45β/CDC2 association in the CuE‐treated GBM8401 and U‐87‐MG cells. Thus, the results indicated that CuE may induce cell cycle delay in the G2/M phase via dissociation of the cyclin B1/CDC2 complex and up‐regulation of GADD45β gene expression.

## 4 DISCUSSION

Our findings provide experimental evidence indicating that CuE may irreversibly arrest GBM8401 and U‐87‐MG cell growth. Previous studies indicated that CuE could significantly inhibit the growth of breast cancer cells and HL‐60 cells by inducing G2/M cell cycle arrest.^14^, ^22^, ^23^ Moreover, CuE could induce cell cycle arrest at the G2/M phase by altering mitotic spindles in U2OS, MCF7 and HeLa cancer cells.^24^ According to the current study, accumulation of CuE in GBM8401 and U‐87‐MG cells is the major cause leading to both the inhibition of cell proliferation and G2/M phase arrest.

Growth arrest and DNA damage 45 proteins have been implicated in the activation of the G2/M checkpoint.^25^ DNA damage 45 can directly inhibit the activity of cyclin B1/CDC2 kinase by interacting with CDC2.^26^ DNA damage 45β, a member of GADD45 family, is an 18‐kDa protein closely related to GADD45α and GADD45γ, which is associated with cell growth control.^27^, ^28^ DNA damage 45β is also a novel tumour suppressor, the tumour‐blocking function of which was illustrated by reducing cell proliferation and survival.^29^ In the current study, we investigated that CuE blocking cell cycle progression and inducing GADD45 gene expression via association with the CDC2/GADD45 complex and displacement of cyclin B1 from the CDC2/cyclin B1 complex in Figure 3. Our finding indicated that CuE in the explored cancer cells led to down‐regulation of the association of the cyclin B1/CDC2 complex and the up‐regulation of GADD45β. Moreover, GADD45β exhibited its binding to the CDC2/cyclin B1 complex, and then regulates the G2/M phases. Previous evidence suggested that the binding of CDC2 with GADD45γ^30^ and Gadd45 family genes is essential for the regulation of antitumour immune responses.^31^ DNA damage 45 gene family participates in cell cycle control, and each of the GADD45 genes may play similar, but not identical, roles in tumorigenesis. Decreased gene expression of GADD45‐α and ‐γ, but not ‐β, were detected in 138 gastric cardia adenocarcinoma (GCA) tissues.^32^ The c‐Jun N‐terminal kinase/stress‐activated protein kinase (JNK/SAPK), encoded by three genes, were responsive to UV irradiation and heat shock.^33^ Sustained activation of JNK is usually thought to be involved in inflammation, apoptosis and proliferation.^34^ DNA damage 45β is important for ERK, p38 and JNK signalling.^35^ DNA damage 45β could inhibit tumour necrosis factor α‐induced JNK signalling via interaction with the JNK kinase, MKK7/JNKK2.^36^, ^37^ However, the expressions of ERK and JNK were not changed significantly following CuE treatment (Figure S2D). Our results indicated that the mitosis delay by CuE is through up‐regulating GADD45β, rather than down‐regulating the gene expression and phosphorylation of ERK and JNK, in GBM8401 and U‐87‐MG cells. Our experimental evidence indicates that the accumulation of CuE in cancer cells could inhibit proliferation and irreversibly arrest the growth of brain cancer cells.

In summary, in this study, efforts were made to determine that CuE is an effective inhibitor of brain cancer cell lines medicated by GADD45β. The mechanism of CuE involvement in the inhibition of tumour growth was highlighted by the delay of mitosis via the up‐regulation of GADD45β expression. The present results speculate the appropriateness of CuE as a potential anticancer agent for the chemoprevention of brain cancer.

## CONFLICT OF INTEREST

The authors declare no conflict of interest.
