# Supplementary figures and images for: The effects of cucurbitacin E on GADD45β‐trigger G2/M arrest and JNK‐independent pathway in brain cancer cells
Source: J Cell Mol Med. 2019 Mar 25;23(5):3512–9. doi: 10.1111/jcmm.14250 (PMC6484297; doi:10.1111/jcmm.14250)

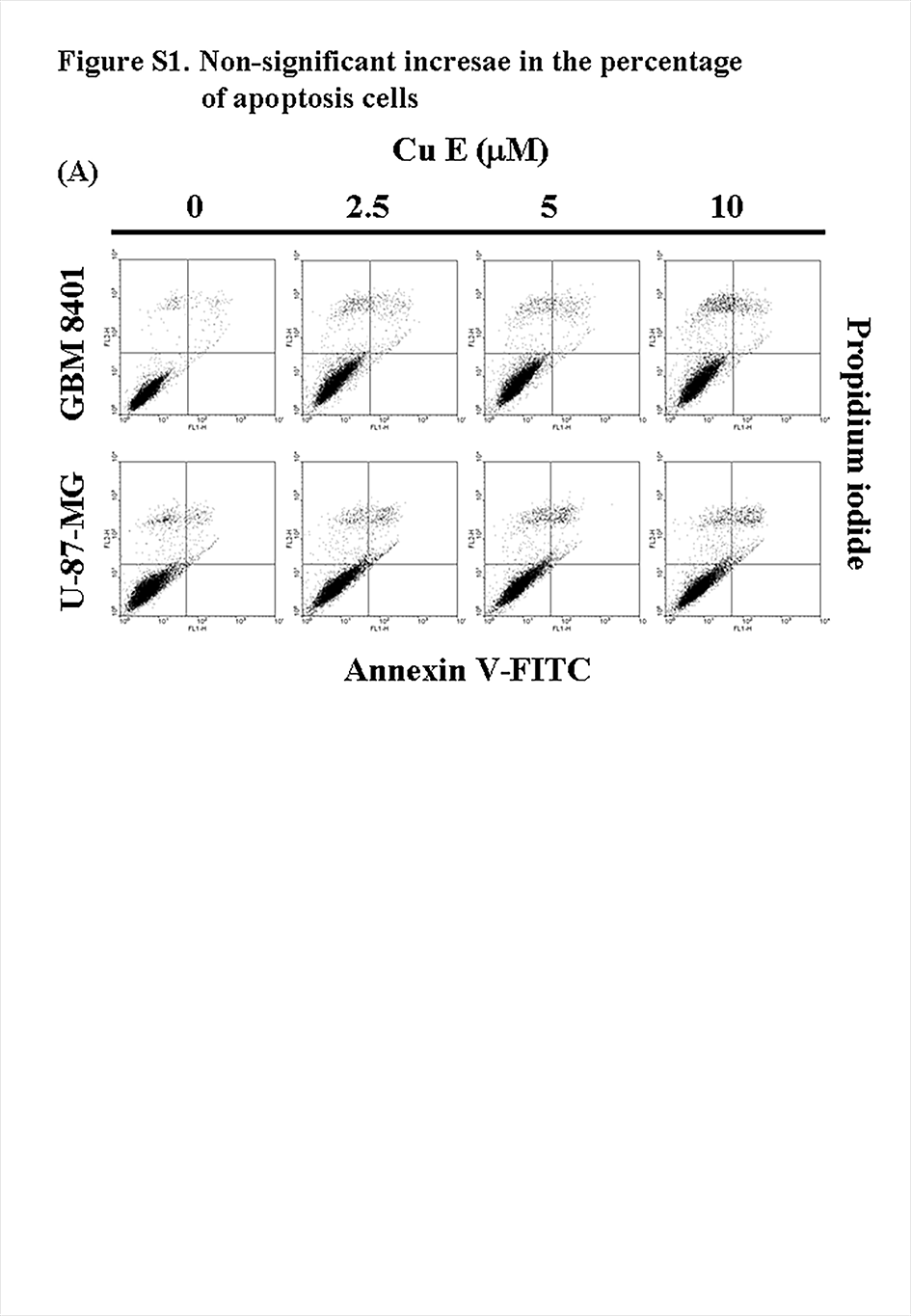

Supplement: Supplementary file 1 [file JCMM-23-3512-s001.tif]

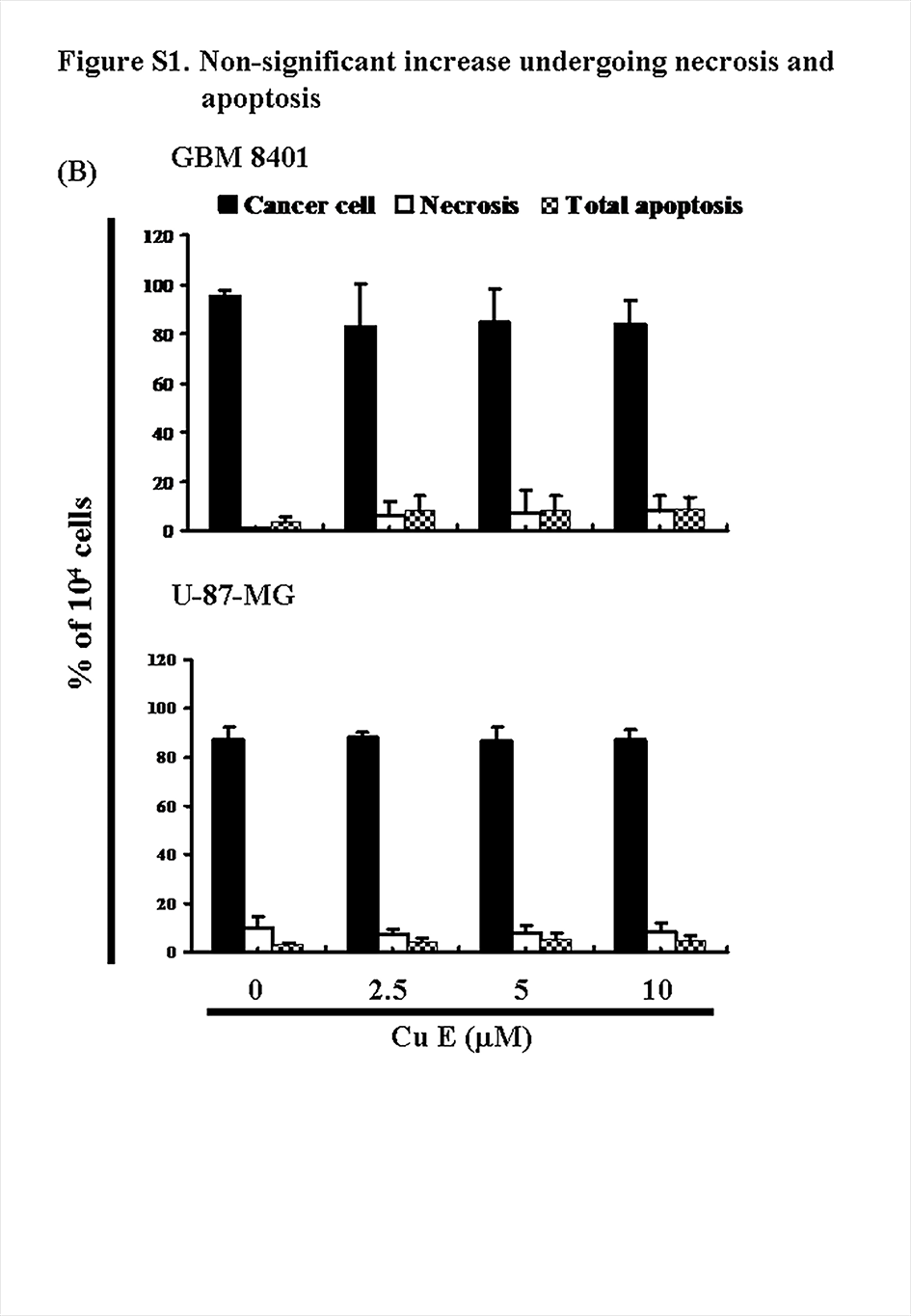

Supplement: Supplementary file 2 [file JCMM-23-3512-s002.tif]

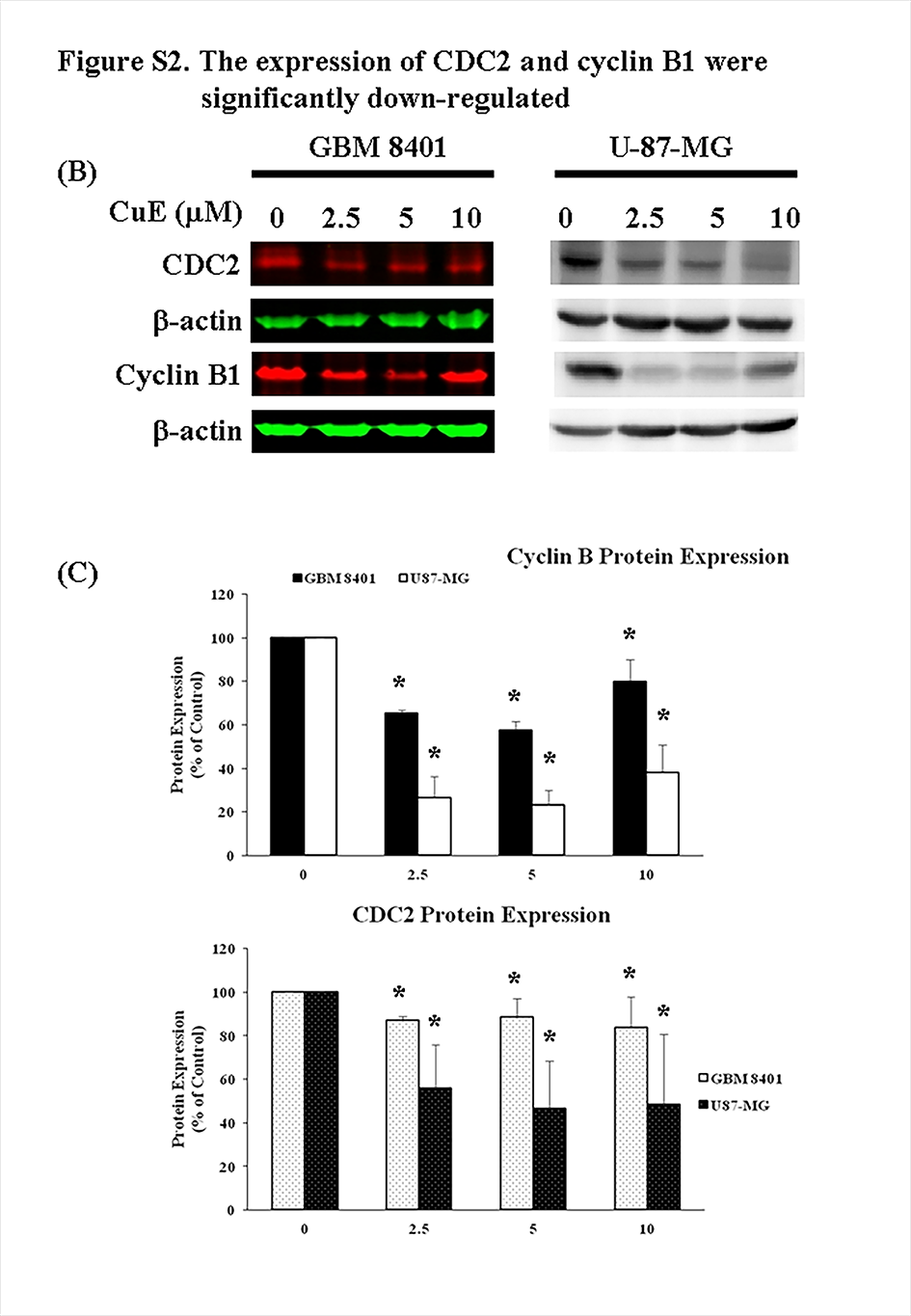

Supplement: Supplementary file 3 [file JCMM-23-3512-s003.tif]

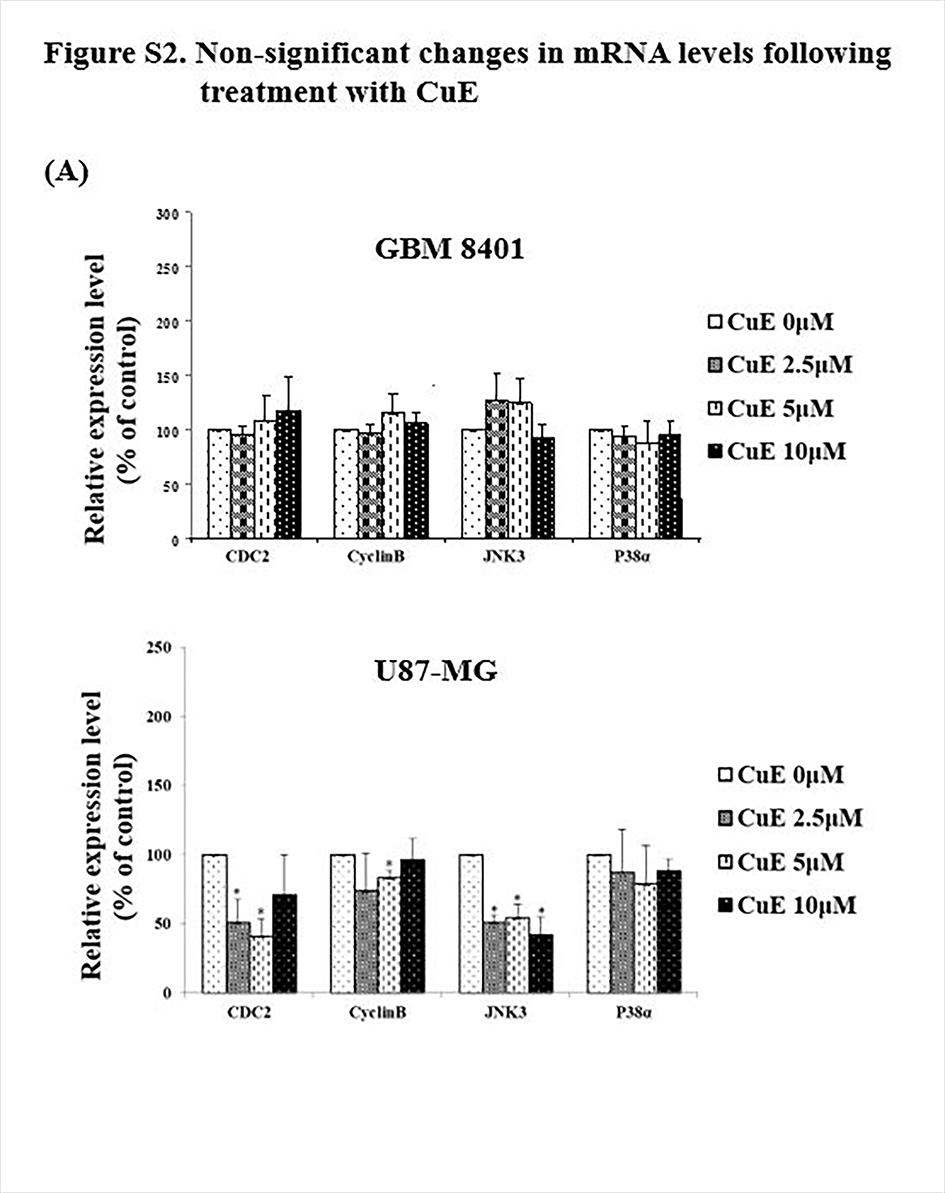

Supplement: Supplementary file 4 [file JCMM-23-3512-s004.tif]

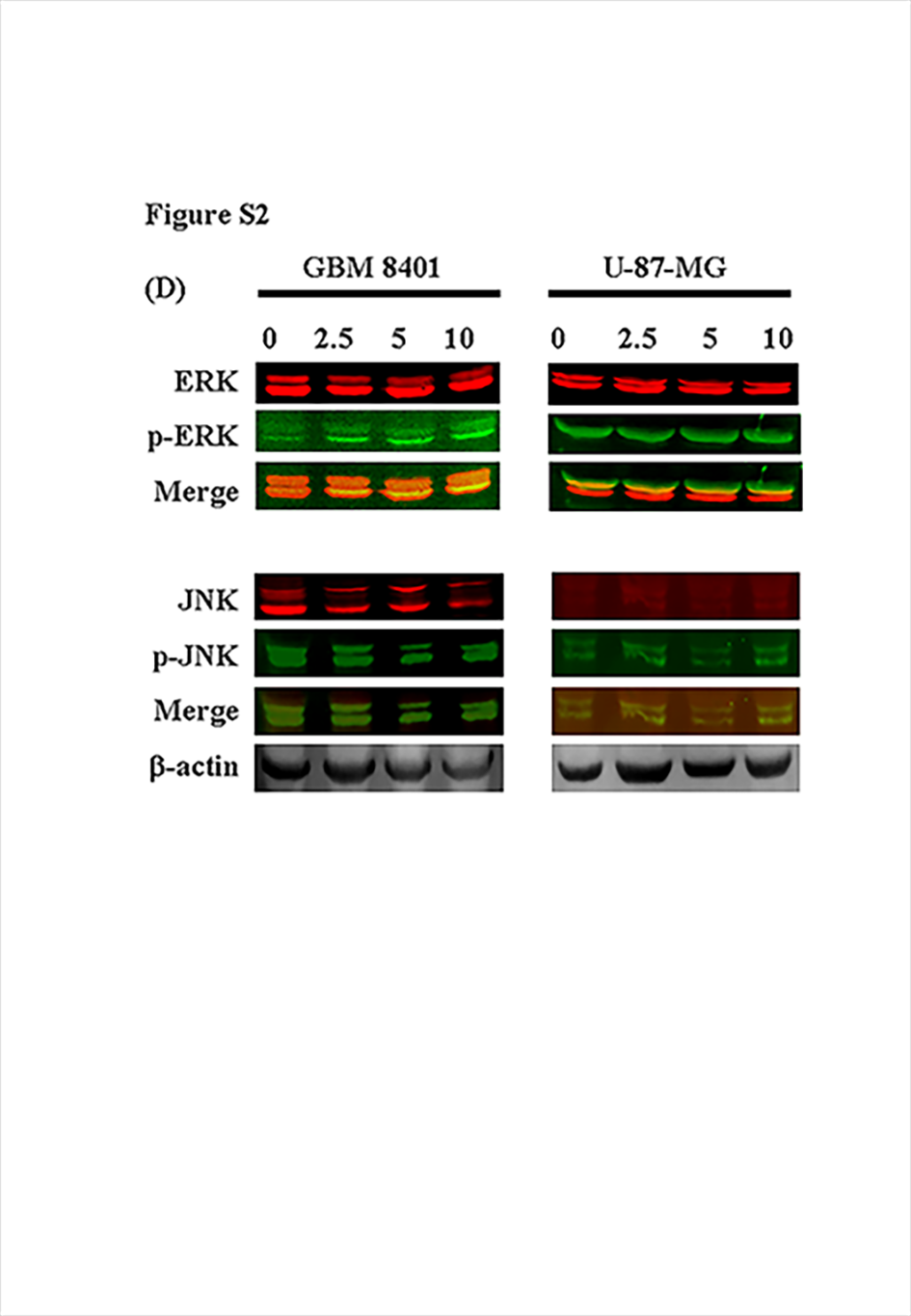

Supplement: Supplementary file 5 [file JCMM-23-3512-s005.tif]
