# Supplementary material for: The effects of cucurbitacin E on GADD45β‐trigger G2/M arrest and JNK‐independent pathway in brain cancer cells
Source: J Cell Mol Med. 2019 Mar 25;23(5):3512–9. doi: 10.1111/jcmm.14250 (PMC6484297; doi:10.1111/jcmm.14250)
Supplement: Supplementary file 6 [file JCMM-23-3512-s006.docx]

**Figure S1.** (A) The effect of CuE on apoptosis/necrosis in the GBM8401 and U-87-MG cells. (B) Total apoptosis of the GBM8401 and U-87-MG cells following incubation with CuE for 4 h. All data are reported as the mean (± SEM) of three separate experiments.

**Figure S2.** CuE mediated ERK and JNK-independent anti-proliferation in GBM8401 and U-87-MG cells. (A) The mRNA levels of CDC2, cyclin B, JNK and p38 which were determined by qPCR. (B) The protein levels of CDC2, cyclin B in the cancer cells following treatment with CuE, as determined though Western blotting and (C) Representative blots from three independent experiments and quantification of band intensities. (D) The protein levels of p-JNK, JNK, p-ERK and ERK in the cancer cells following treatment with CuE, as determined though Western blotting. All data are reported as the mean (± SEM) of seven separate experiments. Statistical analysis was performed using the t test, with differences considered significant at a level of *P < 0.05 versus the 0 μM CuE control group.
